# Fighting malaria in Madhya Pradesh (Central India): Are we loosing the battle?

**DOI:** 10.1186/1475-2875-8-93

**Published:** 2009-05-07

**Authors:** Neeru Singh, Aditya P Dash, Krongthong Thimasarn

**Affiliations:** 1 Regional Medical Research Centre for Tribals and National Institute of Malaria Research Field Station Jabalpur 482003, Madhya Pradesh, India; 2 National Institute of Malaria Research 22 Sham Nath Marg, Delhi 110054, India; 3 WHO SEARO, World Health House, Indraprastha Estate, Mahatma Gandhi Marg, New Delhi 110002, India

## Abstract

Malaria control in Madhya Pradesh is complex because of vast tracts of forest with tribal settlement. Fifty four million individuals of various ethnic origins, accounting for 8% of the total population of India, contributed 30% of total malaria cases, 60% of total falciparum cases and 50% of malaria deaths in the country. Ambitious goals to control tribal malaria by launching "Enhanced Malaria Control Project" (EMCP) by the National Vector Borne Disease Control Programme (NVBDCP), with the World Bank assistance, became effective in September 1997 in eight north Indian states. Under EMCP, the programme used a broader mix of new interventions, i.e. insecticide-treated bed nets, spraying houses with effective residual insecticides, use of larvivorous fishes, rapid diagnostic tests for prompt diagnosis, treatment of the sick with effective radical treatment and increased public awareness and IEC. However, the challenge is to scale up these services.

A retrospective analysis of data on malaria morbidity and associated mortality reported under the existing surveillance system of the Madhya Pradesh (Central India) for the years 1996–2007 was carried out to determine the impact of EMCP on malaria morbidity and associated mortality. Analysis revealed that despite the availability of effective intervention tools for the prevention and control of malaria, falciparum malaria remains uncontrolled and deaths due to malaria have increased. Precisely, the aim of this epidemiological analysis is to draw lessons applicable to all international aid efforts, bureaucracy, policy makers and programme managers in assessing its project performance as a new Global Malaria Action Plan is launched with ambitious goal of reducing malaria and its elimination by scaling up the use of existing tools.

## Background

Madhya Pradesh (Central India) is situated in the central part of India with an area of 308 thousand km^2 ^of which forest covers 76,429 km^2 ^(about 25% of the total land area). According to an estimate made in 1997, 60,35 million population of Madhya Pradesh (MP), accounting for 6% of the total population of India (1,028 million), contributes to 8.6% of the total malaria cases [1]. Malaria is complex in MP because of vast tracts of forest with tribal settlement (20% of state population) [2]. The magnitude of the problem can be assessed from an estimate made in 1987, that 54 million individuals of various ethnic origins residing in forested areas of India and accounting for 8% of the total population contributed 30% of total malaria cases, 60% of total falciparum cases and 50% of malaria deaths in the country [3]. Thus, tribal malaria control requires specific approaches and control strategies [4]. In view of this, the "Enhanced Malaria Control Project" (EMCP) or Tribal Malaria Action Plan was introduced in 1997 by the National Anti-Malaria Programme (NAMP) (now named the National Vector-Borne Disease Control Programme, NVBDCP) with the World Bank assistance, which became effective in September 1997 [5]. The Government of India sought and received a $165 million credit from the World Bank in 1997 to implement the EMCP in 100 high malaria risk districts in eight north Indian states [6]. The primary goal of EMCP was to enable India's NAMP to make a transition from its unsuccessful eradication strategy to more modern control methods. The programme used clearly defined criteria to ensure better focus on the poor and inaccessible group (such as areas with more than 25% tribal population) and areas with high malaria burden (Annual Parasite Incidence (API) >2 per thousand population and reported deaths). EMCP project mainly benefited the tribal population of the targeted districts of eight states and has the flexibility to divert resources to any needy areas in case of any outbreak of malaria [7]. The project was completed in December 2005 and since then the project continued under sustenance phase [8]. The 4^th ^round GFATM established as a source of additional funds for control of malaria, tuberculosis and HIV/AIDS, has not funded any project in MP as it primarily focused on north-eastern states.

A retrospective analysis of data on malaria morbidity and associated mortality reported under the existing surveillance system of the state for the year 1996 to 2007 was carried out to define the epidemiological trends under revised strategy of NVBDCP [7]. The objective of this review is to highlight the realistic and evidence-based malaria situation in tribal dominated areas under EMCP and non-tribal areas without project support and to evaluate the long-term effects of intensive anti-malarial measures.

## The area, people and the health facilities

Madhya Pradesh is a region of deep valleys, hills and hillocks with thick dense forest. The villages are located off the road and terrain is inaccessible. Most tribal villages are formed of three to eight scattered hamlets and lies in undulating terrain with patches of forest. The villages are sparsely populated (200–1,000 inhabitants per village) and are encircled by a perennial stream which provides numerous breeding sites for mosquitoes throughout the year. Houses are small, made of mud, thatch and bamboo with low doors and windows. Fewer than 50% have electricity (only one point connection per dwelling). Often domestic animals are sheltered in the house.

The inhabitants are mostly illiterate (literacy rate, 41.2%)[9], scantily clothed and work mainly in forest nurseries or on road construction and maintenance. The local economy is forest based, with the villagers subsisting on the products of a primitive agriculture. Few (20–40%) children attend school and all have heavy domestic responsibilities. The ethnic tribes spend most of their time outside the dwellings and sleep on the floor of the verandah or out-of-doors.

## Malariometric retrospective data

There are 50 districts in the state however, only 40 are provided with a District Malaria Officer for data collection and coordinating the anti-malaria activities at the district level. Only microscopically confirmed results of either species of malaria parasite are included in the analysis of epidemiological trends. Mixed infections of *P. vivax *and *P. falciparum *are classified as *P. falciparum*. For quality control, 100% positive blood smears and 10% negative blood smears from each district were re-examined by expert microscopists at the state referral laboratory who were unaware of the previous results. Estimates were based on data pooled overall 12 months a year from all the districts from 1996–2007. These estimates were primarily based on active case detection (where a malaria worker goes into a community and takes a blood smears from suspected malaria cases) with minor contributions from passive case detection (where blood smears were made from suspected malaria cases among patients visiting a health centre or a hospital). These data were complied at the state head quarters as reported by the respective District Malaria Officers.

Entomological surveillance conducted earlier revealed that densities of *Anopheles culicifacies *were very high (05–200 per man hour) throughout the year in most of the villages. This species was incriminated as malaria vector, while *Anopheles fluviatilis *occurs in small number (0–7 per man hour), mainly during post monsoon and autumn months [10].

## Malaria control strategies in tribal vs non-tribal areas

Malaria control is mainly based on two powerful tools, Indoor residual spraying (IRS) using DDT (1 gm/m^2^) for vector control and chemotherapy using chloroquine (CQ). Their use is now very problematic because of increasing resistance against DDT in vectors and against CQ in malaria parasites. The EMCP project helped to shift emphasis to a broader mix of effective interventions, including early diagnosis and prompt treatment (EDPT) by hiring link workers, use of insecticide-treated bed nets (ITNs), selective IRS, as well as environmentally friendly measures, such as larvivorous fishes in vector breeding places. In addition, blister packs of CQ and primaquine (PQ) to improve the quality of treatment, and rapid diagnostic kits (RDTs) in remote areas for on-the-spot diagnosis and treatment were also introduced [5]. In contrast, in non-tribal areas, only DDT is used for vector control, and routine surveillance is carried out without link workers and RDTs and CQ remains the main anti-malarial for treatment.

In all, 3998 malaria link workers were hired under the project since 1997 for surveillance of fever cases and for early detection and prompt treatment (EDPT). The synthetic pyrethroid spray coverage was good since inception (1997–2007). Overall, 94% population (2798928) covered under spray out of 2976086 targeted. The room coverage was 86.80% (1558063/1795009). The DDT spray coverage (1997–2007) was also good [8]. Overall, 90.04% of the targeted population covered (4014763/4458800) and the room coverage was 80.4% (3167039/3937141).

Treated bed-nets distribution gradually increased in phases viz 25,000, 50,000, 100,000 and 1.95000 respectively in 2002,03,04 and 05. Although the insecticide status of the net was difficult to ascertain, however, overall 4.79, 4.625 and 5,077 lakhs mosquito nets were re-impregnated during the years, 2005,06 and 07 respectively with synthetic pyrethroid at 6 monthly intervals. In addition to this community owned bed nets were also treated with insecticide.

In all, 118.48, 64.81, 75.54 and 64.28 lakhs larvivorous fish (Gambusia spp) were introduced during 2004, 05, 06 and 07 into large and small ponds that were identified as breeding places of vectors and maintained in stock ponds in each PHC.

Paracheck^® ^RDTs, HRP2 based antigen detection test, (Orchid Biomedical System, Goa, India) were also introduced in phased manner i.e. 15,500, 7935, 80,000, 1,50,000, 200,000 and 248,000 RDTs respectively in 1999, 2002, 03, 04, 05, 06 and 07 for falciparum malaria [11].

## Situation analysis of malaria in Madhya Pradesh

**Figure 1:**
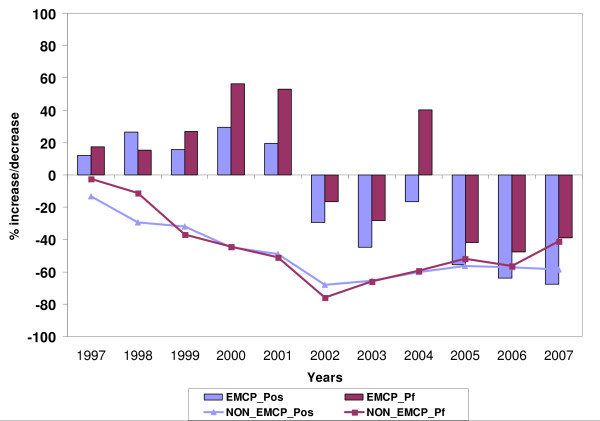
**Percent increase/decrease in malaria and *P. falciparum *cases in districts under EMCP Vs Non EMCP during 1997–2007 (based on year 1996)**.

For this analysis year wise state VBDCP surveillance data was undertaken [8], which showed clear and measurable impact in districts where the World Bank supported malaria control project (EMCP) was implemented. The project was initially launched in 18 districts in MP in 1997 (covering 90 Primary Health Centres [PHCs]) and extended to 20 districts (covering 95 PHC). From 1997 onwards, the year after its pledge to control malaria, a gradual increase in number of malaria infections was recorded upto 2001 (Table 1). The highest numbers of malaria cases were recorded in 2000, when overall malaria increased by 29% when compared with 1996 (Figure 1) and so was *P. falciparum *(56%). Thereafter, there was a moderate decline in number of malaria cases in 2002 (30%) and in 2003 (45%), and in *P. falciparum *infections as compared to 1996 (17 and 28% respectively). However, the decline in the malaria cases, particularly *P. falciparum*, could not be maintained and, by 2004, it appeared to back-track with *P. falciparum *increasing by 40%, while overall malaria showed only a marginal reduction (16%) as against 1996. This was followed by an impressive decline in number of malaria infections in 2005, 2006 and 2007 (Figure 1). However, the proportion of *P. falciparum *cases increased from 33% in 1996 to 62% in 2007. The same scenario was observed in mortality, with the number of deaths increasing from 6 to 35 (6-fold increase) from 1996 to 2006 (Table 2). All fatal cases reported were due to *P. falciparum*, based on blood slide examination, perhaps because of many focal outbreaks each year. For instance, explosive outbreaks were recorded in Chhindwara, Betul, Sidhi, Jhabua and Jabalpur Districts causing many deaths [12-16], thereby putting considerable stress on health service providers. Consequently, the number of deaths reduced to 27 in 2007 [8].

**Table 1:** Records of State Vector Borne Disease Control Programme showing epidemiological trends in EMCP and Non-EMCP districts from 1997–2007 in Madhya Pradesh

**Year**	**Non-EMCP Districts**				**EMCP Districts**			
	**Malaria†**	**(%)**	***P. falciparum *(%)**	***P. vivax***	**Malaria†**	**(%)**	***P. falciparum *(%)**	***P. vivax***
	**BSE‡**				**BSE‡**			
1996	152,728	(2.85)	33,087 (21.66)	119,641	85,494	(5.74)	28,018 (32.77)	57,476
	5,355,899				1,489,417			
1997	132,327	(2·41)	32,159 (24.30)	100,168	95,929	(5·88)	32,947 (34.35)	62,982
	5,489,719				1,632,245			
1998	108,029	(1·85)	29,339 (27.16)	78,690	108,098	(6·27)	32,360 (29.94)	75,738
	5,827,972				1,724,462			
1999	104,103	(1·77)	21,077 (20.25)	83,026	99,146	(4·97)	35,355 (35.66)	63,791
	5,870,745				1,994,498			
2000	8,4051	(1·40)	18,446 (21.95)	65,605	110,638	(4·79)	43,782 (39.57)	66,856
	6,001,517				2,309,468			
2001	78,136	(1·27)	16,169 (20.69)	61,967	102,205	(4·16)	42,876 (41.95)	59,329
	6,140,609				2,459,078			
2002	48,558	(0·80)	8,030 (16.54)	40,528	60,260	(2·37)	23,324 (38.71)	36,936
	6,104,592				2,547,918			
2003	52,591	(0·83)	11,242 (21.38)	41,349	47,117	(1·78)	20,061 (42.58)	27,056
	6,357,722				2,645,959			
2004	60,656	(0·95)	13,497 (22.25)	47,159	71,438	(2·65)	39,270 (54.97)	32,168
	6,375,705				2,600,782			
2005	66,289	(1·01)	15,977 (24.10)	50,312	38,028	(1·54)	16,246 (42.72)	21,782
	6,541,332				2,476,994			
2006	65,354	(0·91)	14,432 (22.08)	50,922	30,776	(1·20)	14,621 (47.51)	16,155
	7,162,466				2,573,432			
2007	63,316	(0.89)	19,497 (30.79)	43,819	27,513	(1.29)	17,125 (62.24)	10,388
	7,038,113				2,131,274			

EMCP – Enhanced Malaria Control Programme.

† Number of blood smear positive for malaria.

‡ Number of blood smear examined from fever cases and cases with history of fever.

**Table 2:** Death due to Malaria in EMCP and non-EMCP districts in Madhya Pradesh (1996–2007)

**Year**	**1996**	**1997**	**1998**	**1999**	**2000**	**2001**	**2002**	**2003**	**2004**	**2005**	**2006**	**2007**
EMCP	6	5	2	7	92	57	26	17	34	41	35	27
Non-EMCP	7	3	1	2	1	4	4	5	2	13	9	14
Total	13	8	3	9	93	61	30	22	36	54	44	41

*Source*: State VBDCP, Bhopal

For districts not under the project, a steady declining trend in number of malaria (range 13–68%) and *P. falciparum *infections (range 3–76%) from 1996 onwards was also recorded. The proportion of *P. falciparum *has held steady (around 22%) from 1996 to 2006 however increased to 31% in 2007. Though there was a dramatic reduction in reported malaria morbidity, deaths due to malaria increased from 7 in 1996 to 14 in 2007 (2-folds increase), mainly because of frequent occurrence of outbreaks. For instance the outbreak in Panna [17] Satna and Shivpuri districts [15] were mainly due to complete cessation of vector control activities and the neglect of active surveillance for a long time.

## Incidence gap and limitations

The EMCP achievements are commendable and unique as there was a sharp steady decline in number of malaria infections in MP from 2002 onward. MP is now contributing only about 5% malaria cases [1]. According to Sarbib *et al *[5], despite the higher burden, reported malaria cases declined much faster in these project districts than in India as a whole. However, the aggregated state level data do not tell the correct trend as would be expected in a site-by-site review of reported district level data. For example Sidhi district which was under EMCP showed a marked deterioration year after year (Table 3). Further, it is worthwhile to mention here that studies conducted by National Institute of Malaria Research (NIMR) field station at Jabalpur district (under EMCP) revealed that in Jabalpur Govt. Medical College alone, 30–80% of indoor patients admitted having complicated malaria of which 20–30% subsequently died (Table 4) due to cerebral malaria (unpublished records of Govt. Medical College Jabalpur). However, these deaths are not shown in the records of State Health/NVBDCP. Thus, despite the World Bank initiative, the overall malaria risk remains stable, although cases may appear to have shown a declining trend. These results are in line with results from Orissa, the adjacent highest malarious state, contributing 23% malaria, 40% *P. falciparum *and 50% of malaria deaths of the country even after the implementation of EMCP [18]. This would be further strengthened by the fact that the reported malaria incidence in India from 1990s to date has been around 1·5 to 2·6 million cases and 666–1,000 deaths/year (NVBDCP, unpublished records), whereas estimated incidence by the WHO was 15 million malaria cases with 19,500 to 20,000 deaths/year [19]. Furthermore, in a recent study in Gujarat state in western part of India, it was estimated that there were on an average 25,465 malaria cases/year as against 4,119 cases reported and at least 22 malaria deaths/million populations, as against 0.3/million reported [20]. Thus, there existed glaring gaps between the reported and the true burden of malaria in India.

**Table 3:** Epidemiological data of Sidhi district of Madhya Pradesh (1996–2007)

**Year**	**Malaria †**	**(%)**	***P. falciparum *(%)**	***P. vivax***	**Deaths**
	**BSE‡**				
1996	2,033	(1.51)	416 (20.46)	1,617	0
	134,535				
1997	1,203	(0·80)	378 (31.42)	825	0
	150,897				
1998	1,000	(0·63)	275 (27.50)	725	0
	158,007				
1999	1,223	(0·67)	422 (34.50)	801	0
	182,648				
2000	2,071	(1·09)	561 (27.09)	1,510	33
	190,024				
2001	2,179	(1·05)	882 (40.48)	1,297	9
	208,026				
2002	2,098	(1·07)	620 (29.55)	1,478	3
	196,630				
2003	3,504	(1·85)	1172 (33.45)	2,332	1
	189,350				
2004	3,004	(1·52)	936 (31.16)	2,068	8
	197,371				
2005	5,192	(2·46)	2,180 (41.99)	3,012	25
	211,356				
2006	7,761	(2·77)	3364 (43.34)	4,397	25
	280,238				
2007	5,833	(2.39)	3,104 (53.21)	2,729	1
	243,680				

*Source*: District Malaria Officer Sidhi

† Number of blood smear positive for malaria.

‡ Number of blood smear examined from fever cases and cases with history of fever.

**Table 4:** Records (2000 – 2007) of indoor admission at Govt. Medical College Jabalpur, Madhya Pradesh showing morbidity and mortality due to Cerebral Malaria (CM)

**Year**	**Total Malaria Admission**	**Total In-patient Admission for Cerebral Malaria (%)**	**Deaths due to Cerebral Malaria (%)**
2000	217	112 (51·6)	32 (28·5)
2001	237	155 (65·4)	50 (32·2)
2002	113	90 (79·6)	23 (25·5)
2003	91	60 (65·9)	20 (33·3)
2004	75	48 (64·.0)	9 (18·75)
2005	109	68 (62·3)	12 (17·6)
2006	100	34 (34·0)	7 (20·5)
2007	135	59 (43.7)	19 (32.2)

## Why malaria control failed?

Review of the surveillance data and an analysis of the relationship between morbidity and potential mortality from malaria suggest that no intervention aiming to reduce the malaria burden was set-up on a long-term basis in most of the epidemiological settings. A number of technical and operational issues related to intervention tools are discussed in detail for better understanding and strategic support.

### Targeted interventions

Interventions were highly targeted to malaria high-risk PHC's (predominantly tribal) in most districts and ignoring the other high malaria burden PHC's and affected non tribal districts, although overall malaria transmission intensity is moderate to low in most of these districts, and each had pockets of high level transmission whether in EMCP or not. Health infrastructure in these districts is weak and the population are not well connected from public health structures. For example in Sidhi district which has eight PHC's and all are located in very remote with inaccessible terrains, only two brought under the EMCP and interventions were restricted to these PHC's only. Consequently, remaining six PHC's are in the grip of malaria (Table 3). The gravity of the problem can be assessed from the fact that in Vaidhan PHC, (not under EMCP) in the first week of June 2007, 13 patients with fever were admitted in Community Health Centre (CHC) hospital, all showed heavy *P. falciparum *infection and two died. The month of June is not the transmission season for *P. falciparum*. Likewise, in Jabalpur, out of eight PHCs only one was under EMCP. Such targeting of intervention is only effective if the malaria surveillance system reliable, laboratory capacity skilled, case reporting accurate and all information is computerized and readily available.

## Drug resistance and chemotherapy

The distribution of *P. falciparum *malaria and patterns of its resistance to CQ are not homogeneous. There is no regular system to evaluate the parasite sensitivity to malaria drugs and to update the ministry and implementation agencies in line with the changing situation within various part of state. On the basis of available record, the programme continued to use CQ as first line drug because *Plasmodium vivax *causes about 52% of the country's confirmed malaria cases. Thus, despite global pressure to adopt artemisinin-based combination therapy (ACT), NVBDCP achieved success by focusing on scaling up rapid treatment with CQ, Blister pack and SP [6]. In reality, *P. falciparum *has evolved resistance to CQ in most districts of the state, either partially or completely, and, as a result, *P. falciparum *has taken deep roots causing high morbidity and mortality [21]. Therefore, the NVBDCP has decided to switch from CQ to a blister pack ACT (sulphadoxine-pyrimethamine + artesunate) at a country-wide scale in phases since 2008–09.

## ITNs

ITNs have proved to be a highly effective control tool provided that people use them properly and consistently and coverage is high. However, ITNs were procured in very small quantity and the distribution pattern was not clear, i.e. who are the beneficiaries, targeted group and percent coverage. Further, EMCP started in 1997 and the available records showed bednet distribution from 2002 onwards. Furthermore, a household survey reported a low ITN coverage rate and poor upkeep [22]. LLINs have not been introduced in MP.

## RDTs

Like wise RDTs which are being used for strengthening EDPT by on-the-spot diagnosis and treatment in remote areas [23], are procured only in tiny quantity. Consequently, malaria is diagnosed clinically on the basis of fever or by microscopic examination of blood smears at PHC level. Further, necessary training regarding RDTs, their handling and use are not provided in any district, and, as a result, RDTs are either not used or used at district hospital and in doctor's clinics.

## Limited human resource capacity

There is a human resource crisis in the targeted districts. With a high disease burden it is an enormous challenge to ensure that key interventions are delivered timely as link workers through which EDPT was made possible are not sufficient in number for optimal performance. This results in an overburdening of health care staff, which coupled with low financial remuneration, leads to low moral and poor performance.

## Conclusion and recommendations

This review has attempted to provide important and hitherto unreported insights about tribal malaria [2]. Systematic disease surveillance is essential to decision maker, but no such systematic surveillance data exists for malaria. Free services at health facilities do not remove other barriers to reaching a clinic, such as lack of transportation and frequent power cuts at the clinic (which prevent the microscopic examination of blood smears). What appears to have been most important was the full-scale implementation of EDPT using RDTs and ACTs. Without high quality data it would be impossible to monitor progress and focus efforts on malaria control on sustainable basis. Further, there should have been capacity to analyse and interpret surveillance data accurately as with the existing capacity the state malaria officers may not be able to locate the risk areas and identify risk population. Weak infrastructure did prevent the programme manager from taking control interventions to scale. Furthermore, although the programme acknowledges that EDPT is essential to reduce malaria death, RDTs are not being used systematically in the absence of proper training. Therefore, it is very important that the operational costs of implementing a policy change (development of guidelines and other implementation tools, training and supervision) are not overlooked during financial planning. There is, therefore, a strong case for enhanced funding for malaria control. A quantum jump in funding can only save the situation from emerging challenges of tribal malaria. The use of GFATM resources to supplement malaria control in tribal belt of MP would be ideal. If these issues are not addressed quickly in the proposed new phase World Bank-funded Vector Borne Disease Control Programme, then success is unlikely.

Since conditions for the occurrence of outbreaks are very favourable, epidemic forecasting systems have to be established. Reporting is to be done on a weekly basis and districts must be expected to report any increase in the number of malaria cases. Since malaria parasite prevalence surveys are costly, the use of serology for the assessment of exposure to malaria in population might give reasonable estimates [24]. Depending on the level of exposure, an appropriate package of intervention could then be implemented in the targeted areas [25]. Regular monitoring is also essential to protect intervention tools. Resistance to the insecticide DDT and the treatment with CQ have contributed to the abandon of the malaria eradication campaign in the 1960s. Today's efforts also depend on two main tools, i.e. vector control using pyrethroids for indoor residual spraying or to treat bed nets and artemisinin derivatives for treatment [26]. Resistance has already been reported for the former [27] and emerging for the latter [28]. The target of malaria control goals would fail if detection and response to resistance remain inadequate.

Finally, to maximize the effectiveness of limited resources, budgetary provision should have been kept for trainings, and capacity building (Telephones, Fax, Computers, Vehicles and Ambulance). The increased use of computer technology would reduce dependence on hand-written registers, the completion of which represents an onerous and time-consuming task for health staff. The two funding agencies (GFATM and World Bank) should ideally work together to increase synergies to reach the ambitious goal of Global Malaria Action Plan [29] to reduce malaria burden and deaths by 2015 and moving towards the elimination of malaria. Clearly, more effective measures must now be taken to contain the transmission of malaria.

## Competing interests

The authors declare that they have no competing interests.

## Authors' contributions

All authors provided critical review of the text and approved the final version.
